# Vitamin B12 Status of Various Ethnic Groups Living in New Zealand: An Analysis of the Adult Nutrition Survey 2008/2009

**DOI:** 10.3390/nu10020181

**Published:** 2018-02-07

**Authors:** Asika Devi, Elaine Rush, Michelle Harper, Bernard Venn

**Affiliations:** 1 Department of Human Nutrition, University of Otago, P.O. Box 56, Dunedin 9054, New Zealand; asika.d10@gmail.com (A.D.); michelle.harper@otago.ac.nz (M.H.); 2 School of Sport and Recreation, Auckland University of Technology, PB 92006, Auckland 1142, New Zealand; elaine.rush@aut.ac.nz; 3 Riddet Institute, Massey University, Private Bag 11222, Palmerston North 4442, New Zealand

**Keywords:** vitamin B12, national survey, dietary intake, ethnicity

## Abstract

Vitamin B12 deficiency leads to serious health problems, whilst sub-optimal status is associated with raised biochemical markers of disease risk. Identifying at-risk groups could benefit both individuals and public health. Dietary data were sourced from the New Zealand Adult Nutrition Survey 2008/2009, involving a nationally representative sample of 4721 participants. Ethnic groupings were by regional origin: Māori and Pacific Islands, New Zealand European, East and South-East Asian, and South Asian. Diets were assessed using 24-h recalls and from responses to a questionnaire. Blood samples were obtained from a subset (*n* = 3348). The mean (95% CI) vitamin B12 intake of the Māori and Pacific Islands group was 5.1 (4.7, 5.5) µg/day, New Zealand Europeans 4.1 (3.8, 4.3) µg/day, East and South-East Asians 4.5 (3.7, 5.3) µg/day, and South Asians 3.0 (2.5, 3.6) µg/day. Overall, 20.1% of the sample had vitamin B12 inadequacy (<221 pmol/L). South Asians had the lowest vitamin B12 concentration at 282 (251, 312) pmol/L, whilst Māori/Pacific and East/South-East Asians had the highest, at 426 (386, 466) and 425 (412, 437) pmol/L, respectively. The main dietary determinant of serum vitamin B12 concentration was whether or not people ate red meat, with a regression coefficient of 27.0 (95% CI: 6.6, 47.5). It would be helpful for health agencies to be aware of the potential for compromised vitamin B12 status in South Asian communities.

## 1. Introduction

Vitamin B12, also known as cobalamin, is a water-soluble B-vitamin [1]. Vitamin B12 is synthesized by bacteria, and finds its way into the human food supply predominantly via animal products [2]. This places low consumers of animal products—vegetarians, and particularly vegans—at risk of low intake [3] and metabolic deficiency [4]. Other causes of deficiency are caused by malabsorption of the vitamin due to tropical sprue [5], *Helicobacter pylori* [6], hypochlorhydria [7], and gastric atrophy [8]. Human deficiencies of vitamin B12 result in adverse effects to the individual that, when prevalent in the population, may be of public health significance [9]. The clinical manifestations of vitamin B12 deficiency result in haematologic, neurologic, psychiatric, and other chronic illnesses [10]. Breastfeeding infants of vitamin B12-deficient mothers are at particular risk of potentially life-threatening complications if the deficiency is not identified and rectified [11]. The prevalence of frank deficiency resulting in haematological or neurological problems is relatively uncommon, but subclinical deficiency has been estimated to range from 2.5% to 26% in various populations [12]. Functional markers of vitamin B12 status, namely methylmalonic acid and homocysteine, are elevated when vitamin B12 status is deficient (<148 pmol/L) or suboptimal (<221 pmol/L) [12]. The functional biomarkers may also be raised when vitamin B12 status is between 148 and 221 pmol/L. This sub-optimal status may confer additional risk for neurodegenerative disease [13,14] and for women, neural tube defects in their offspring [15].

Areas identified as having populations at risk of low vitamin B12 intakes include the Indian subcontinent [16], Central and Southern America, and parts of Africa [17]. Whether people who emigrate from these areas are similarly susceptible to low vitamin B12 intakes will be of interest to their new country. People of Indian and Pakistani descent living in Toronto were found to have a high prevalence of vitamin B12 deficiency, attributed to a low intake of animal products, compared with the deficiency rates in the general population [18]. New Zealand has immigrant populations from across Asia, with both vitamin B12 intakes and biochemical data available for analysis from the New Zealand Adult Nutrition Survey, a survey conducted on a nationally representative sample in 2008/2009. Given the previous research, we hypothesize that people of South Asian origin living in New Zealand will have lower vitamin B12 intakes and status compared with other immigrant and indigenous groups. We further hypothesize that cultural and religious practices will predispose South Asian people to low intakes of animal products. Data collected in 1997 from a previous national nutrition survey suggest that a high proportion of older New Zealanders were at risk of vitamin B12 insufficiency among a predominantly European sample of adults [19]. Analysis of data from the current New Zealand Adult Nutrition Survey provides an opportunity to assess vitamin B12 intakes and determinants of biochemical status among a unique set of ethnic groups across a wider age range. Thus, the primary aims of the study are to compare vitamin B12 intake and status among ethnic groups living in New Zealand, with a secondary objective to assess dietary factors associated with intake and status.

## 2. Materials and Methods

The New Zealand Adult Nutrition Survey was a nationally representative, cross-sectional survey of 4721 New Zealanders aged 15 years and above. The New Zealand Health and Disability Multi-Region Ethics Committee granted ethical approval for the survey (MEC/08/04/049). A full account of the methodology has been published [20]. In brief, participants were recruited using a three-stage, stratified, area-based sampling frame involving 607 geographical areas, followed by the selection of households within each area and a randomly selected respondent within each household. Informed, written consent was obtained from the participants. The survey was undertaken from October 2008 to October 2009 and had a response rate of 61%.

### 2.1. Data Collection: Dietary, Anthropometric, and Lifestyle

During a home visit, a trained interviewer assessed weight, height, and waist circumference using calibrated instruments, and collected socio-demographic and dietary information using proprietary computer-assisted interview software. An indicator of socioeconomic status of participants was attained via the New Zealand Index of Deprivation 2006 [21]. Dietary information was via a four-stage multi-pass 24-h diet recall and a dietary habits questionnaire. The four stages of the 24-h recall were as follows:

(a) A “quick list” of all foods, beverages, and dietary supplements consumed during the preceding day (midnight to midnight) was obtained.
(b) Detailed descriptions were obtained of all items consumed, using specific questions and prompts, including cooking method, recipe for mixed dishes (where known), any additions made before consumption, brand and product name, time consumed, and where the food was sourced. Brand and product name were determined using a bar code scanner for food items where the composition was brand specific and packaging was available.
(c) Estimates were made of amounts of items consumed, wherever possible (e.g., cups, tablespoons), using food photographs, shape dimensions, food portion assessment aids (e.g., dried beans), and packaging information.
(d) All items were reviewed in chronological order. Any additions and changes were made at this point.

Information on the nutrient content of foods was obtained from the New Zealand Food Composition Database [22] and nutrient composition calculated using bespoke software developed in-house by the University of Otago Department of Human Nutrition staff [23].

The following four questions were used from the Adult Nutrition Survey Dietary Habits Questionnaire [24]:

1. In the past four weeks which of the following have you eaten at all?

Red meat—such as beef, pork, mutton, lamb and goat.

Chicken—such as chicken breast, drumsticks, or whole chickens, but not chicken nuggets or chicken roll; Processed meats—such as ham, bacon, sausages, luncheon, canned corned beef, pastrami and salami; Seafood—such as fish or shellfish

Responses to these questions were coded Selected (1), Not selected (0).

2. On average how many servings of fruit—fresh, frozen, canned, or stewed—do you eat per day?

Never, I don’t eat fruit; Less than one serving per day; 1 serving; 2 servings; 3 servings; 4 or more servings; Do not know.

3. On average how many servings of vegetables—fresh, frozen, or canned—do you eat per day?

Never, I don’t eat vegetables; Less than one serving per day; 1 serving; 2 servings; 3 servings; 4 or more servings; Do not know.

4. What type of milk do you use the most of?

None, I don’t use milk; Whole or standard milk; Reduced fat; Skim or Trim; Soy milk; Other (such as rice, goats milk); Do not know.

The responses to questions 2 and 3 (fruit and vegetables) were summarized and analysed as a single “fruit and vegetable” variable to assess whether participants were meeting the “5+ a day” recommendation.

The responses to question 4 were dichotomized into cow’s milk drinkers or non-cow’s milk drinkers, to assess the specific contribution of vitamin B12 to the diet from cow’s milk.

A question on smoking (SC2_Q11: How often do you currently smoke?) and one on alcohol intake (SC2_Q16: How often do you have a drink containing alcohol?) were also used in the analysis.

### 2.2. Blood Biochemistry

A non-fasting blood sample was obtained from 3348 non-pregnant survey respondents, giving a response rate of 71% of the total sample. To give a blood sample, participants attended a health clinic in their locality. The blood samples were processed by the local clinics, and aliquots of serum were sent overnight at 4 °C to the University of Otago, where the samples were stored at −80 °C until analysis. There was insufficient blood to complete a vitamin B12 analysis for 276 participants. Vitamin B12 analyses were performed using a chemiluminescent kit (Roche Diagnostics GmbH, Mannheim, Germany) on a Roche Hitachi Elecsys2010 system (Hitachi High-Technologies Corporation, Tokyo, Japan). The within-batch coefficients of variation of a pooled plasma sample and the manufacturer’s control were both <5%. The absolute concentrations of repeated control samples were within 3% of the manufacturer’s stated concentration.

### 2.3. Ethnicity

Ethnicity was self-reported, using the Statistics New Zealand standard ethnicity question from the New Zealand Census 2006. The question was “Which ethnic group do you belong to?”, with tick boxes for New Zealand European, Māori, Samoan, Cook Island Māori, Tongan, Niuean, Chinese, Indian, and Other. People could identify with more than one ethnicity, so for the purpose of this analysis, a single ethnicity was assigned based on pre-determined prioritization. For example, a person was classified as Māori if any one of their recorded ethnicities was Māori. The prioritization order was Māori followed by Pacific Island, which included those people who identified as Samoan, Cook Island Māori, Tongan, Niuean, and Fijian. Other ethnicities were encompassed within the term New Zealand European and Other. South Asian refers to all people who have ancestral origins in the Indian subcontinent (the countries of India, Afghanistan, Pakistan, Sri Lanka, Nepal, Bangladesh, Bhutan, and the Maldives). Fijian Indians were also included in the South Asian category, because their ancestry is from the Indian subcontinent, and it is hypothesized that they brought their cultural habits with them to the new country of residence (the South Asian diaspora) [25]. East and South-East Asian included Chinese, Malaysian Chinese, Taiwanese, Filipino, Cambodian, Vietnamese, Burmese, Indonesian, Japanese, Korean, and Tibetan people. There were 49 people who did not fit into any of the ethnic groupings; these were predominantly of African origin, and their data were not used in the analyses presented here.

### 2.4. Statistics

Survey-prioritized weighting was used in all analyses to ensure that no group was under- or over-represented, such that the estimates of means, percentiles, and proportions were representative of the total resident adult population of New Zealand. Blood-prioritized weight was exclusively used when performing statistical analyses of serum vitamin B12 data. The procedure for weighting is described in detail in the Survey Methodology Report [20].

Multiple linear regression analysis was used to quantify the independent effect of dietary, lifestyle, and socioeconomic exposures on serum vitamin B12. Age, sex, and ethnicity were regarded as confounding variables and were controlled for in the analyses. Pre-specified sub-groupings included grouping by ethnicity and sex. The adult estimated average B12 requirement of 2 µg/day was used to determine adequacy of vitamin B12 intake [26]. The cut-off used to define vitamin B12 deficiency was <148 pmol/L (200 pg/mL), and depletion was defined as 148–221 pmol/L (200–300 pg/mL), as suggested in the literature [27]. There were some exceptionally high vitamin B12 intakes and serum values in the dataset. Extreme values were quantified using an outlier algorithm, in which the inter-quartile range was multiplied by 1.5 and added to the third quartile [28]. Using this algorithm, the serum and intake outlier values calculated were 701 pmol/L and 9.56 µg/day, respectively. Intakes of around 10 µg/day (five times the estimated average requirement) and serum concentrations in excess of 700 pmol/L were more likely the consequence of supplementation or injection than diet alone. As high intakes from supplements and or injections were secondary to the aim, only serum vitamin B12 concentrations values <701 pmol/L and vitamin B12 intakes <9.56 µg/day were included in the regression, resulting in the data of 12 people being dropped. In total, the serum vitamin B12 concentrations of 3011 participants were included in this analysis.

Variables that were not normally distributed were log transformed. Chi-squared and Fisher’s exact tests were used to check for association between categorical variables. Differences among ethnic groups were assessed using one-way ANOVA with Bonferroni multiple-comparison test. Statistical analysis was performed with STATA version 12 (StataCorp LP, College Station, TX, USA).

## 3. Results

### 3.1. Participant Characteristics

The characteristics of the participants are shown in Table 1.

### 3.2. Vitamin B12 Intake and Serum Concentrations among Ethnic Groups

All regional ethnic groups had a mean vitamin B12 intake above the estimated requirement of 2.0 µg/day. Vitamin B12 intakes of South Asians and East and South-East Asians were not significantly different to New Zealand Europeans, with the Māori and Pacific grouping having the highest intake (Table 2). The overall estimated prevalence of inadequate intake for vitamin B12 (i.e., vitamin B12 intake < 2.0 µg/day) was 34%, the prevalence of which was highest among the South Asians. Of those who answered the question on smoking (*n* = 1240 non-smokers; *n* = 1067 current smokers; *n* = 2414 unstated status), the mean vitamin B12 intakes of non-smokers and current smokers were 4.6 (SD 8.5) and 4.9 (SD 6.5) µg/day, respectively.

Serum vitamin B12 concentrations of South Asians were less than New Zealand Europeans, and East and South-East Asians and Māori and Pacific had concentrations of, on average, 100 pmol/litre more than the New Zealand European group. The overall estimated prevalence of vitamin B12 deficiency (serum vitamin B12 < 148 pmol/L) and sufficiency (serum vitamin B12 > 221 pmol/L) was 2% and 80%, respectively. There was a higher prevalence of vitamin B12 insufficiency in New Zealand Europeans (37%) and South Asians (57%), compared with the East and South-East Asians and Māori and Pacific (15%) (Table 2). Of those who answered the question on smoking (*n* = 894 non-smokers; *n* = 567 current smokers; *n* = 1550 unstated status), the mean serum vitamin B12 concentrations of non-smokers and current smokers were 344 (SD 176) and 369 (SD 164) pmol/L, respectively.

### 3.3. Serum Vitamin B12 by Age and Ethnicity

Serum vitamin B12 concentrations were lower overall in the older age groups (65 years and older) compared with the 15–64 years age bracket (Table 3). The only exception was Māori and Pacific, who maintained vitamin B12 concentrations over the 65–75 years age range, showing a decline only after 75 years.

### 3.4. Vitamin B12 Intake and Status Grouped by Dietary Components

Ninety-six percent of New Zealand European, as well as 95% of East and South-East Asians and Māori and Pacific groups consumed red meat. This compares with 69% of the South Asians (*p* < 0.05). Overall, 95% of the study population was milk consumers, but only 91% of East and South-East Asians consumed milk, which was less than for the other ethnic groups (*p* < 0.05). The biggest consumer of fruits and vegetables among the groups were New Zealand Europeans (*p* < 0.05). There were no differences in fruit and vegetable consumption among South Asians, East and South-East Asians, and Māori and Pacific. The mean vitamin B12 intake and serum concentration were higher for red meat-eaters compared with non-red meat-eaters (Table 4). Among milk consumers and non-milk consumers, there were no statistical differences in vitamin B12 intake (*p* = 0.118) or serum vitamin B12 concentrations (*p* = 0.460). There was no difference between vitamin B12 intakes (*p* = 0.058) or serum vitamin B12 concentrations (0.078) between consumers or non-consumers of five or more servings of fruit and vegetable per day.

### 3.5. Vitamin B12 Intake by Food Sources among Ethnic Groups

A high proportion of vitamin B12 for the South Asian participants was provided by dairy, with a low proportion coming from meat (Table 5). In contrast, East and South East Asians and Māori and Pacific participants tended to have a lower proportion of vitamin B12 coming from dairy, and a relatively high proportion from meat, compared with the Caucasian participants. The type of meat also differed among ethnic groups; for example, the South Asians tended to consume less beef and more lamb than the Caucasians.

### 3.6. Predictors of Vitamin B12 Status

Vitamin B12 intake and red meat consumption were positively associated with serum vitamin B12 concentration, and age was negatively associated with serum vitamin B12 (Table 6). There was no association between serum vitamin B12 and fruit and vegetable intake, BMI, socioeconomic status, sex, or consumption of cow’s milk.

## 4. Discussion

Our data indicate variability in the vitamin B12 intakes and biochemical status among residents of New Zealand based on regional ethnic groupings. East and South-East Asians had a similar intake and status to Māori and Pacific, with Māori and Pacific having a higher vitamin B12 intake and status compared with New Zealand Europeans. South Asians had intakes lower than East and South-East Asians and Māori and Pacific, with serum concentrations lower than all other groups. Thus, Māori and Pacific were the least likely to have inadequate intakes, compared with New Zealand Europeans, and the latter group was more likely to have an adequate biochemical status than the South Asians.

These data are consistent with other work. In Canada, a high prevalence of vitamin B12 deficiency was found among South Asian men and women registered with a Toronto clinic [18]. In the United Kingdom, healthy European men had higher vitamin B12 status (357 pmol/L) compared with South Asian counterparts (270 pmol/L) [29]. That value of 270 pmol/L is very comparable to the mean serum concentration of 282 pmol/L in our South Asian group living in New Zealand. A smaller proportion of our South Asian group were above the sufficient cutoff than the other groups, indicating that as a group, South Asians may be more at risk of subclinical deficiency, based on elevated functional markers of vitamin B12 metabolism [30]. Limitations of our work were the absence of such functional biomarkers of vitamin B12 status, namely total plasma homocysteine, methylmalonic acid, and holo-transcobalamin. These functional biomarkers are useful in assessing the likelihood of depletion in the intermediate range of serum vitamin B12 concentration (148–221 pmol/L) [27]. Plasma total homocysteine concentration is also affected by folate status via folate–cobalamin interactions [31]. However, serum vitamin B12 concentration in and of itself is a valid indicator of vitamin B12 status, with a value of 148 pmol/L widely used in the epidemiologic setting to indicate insufficiency [32]. Around 3% of the South Asian and New Zealand European groups were below this cutoff. Overt vitamin B12 deficiency has been associated with megaloblastic anemia in Asian vegetarians [33]. The elderly may also be compromised, with vitamin B12 being inversely associated with cognitive tests of spatial copying skills [34]. Pregnant women with vitamin B12 deficiency and insufficiency are at risk of having a child with birth defects, and low maternal status further puts the infant at risk of frank deficiency and failure to thrive, particularly if the mother breastfeeds [35]. If the low maternal vitamin B12 status is due to an inadequate intake, perhaps for cultural or religious reasons, then it is likely that the child will be raised in a low vitamin B12 environment with longer-term consequences. Low vitamin B12 status in Indian mothers has been associated with poorer scores of cognitive tests in their nine-year-old children [36]. Thus, lower mean vitamin B12 status exposes a higher proportion of South Asian individuals, compared with other ethnic groups, to an elevated risk of metabolic insufficiency, with around 3% of South Asians and New Zealand Europeans at risk of clinical deficiency.

The relatively low vitamin B12 status of South Asians has been attributed to vegetarianism [29]. However, low intakes and status are not exclusively associated with strict vegetarianism, as South Asians may avoid certain meats or consume limited amounts of animal products, due to cultural and religious practices [37]. We did not ask directly about cultural and religious practices, but the relatively high proportion who avoided meat suggested that such practices may be contributing factors to the lower vitamin B12 status of the South Asian group. Indeed, meat eaters had considerably higher serum vitamin B12 concentrations than non-meat eaters, resulting in meat intake being a major predictor of vitamin B12 serum concentrations. A major source of dietary vitamin B12 for South Asians was milk, with 98% of the group consuming it. Despite this uptake, overall intake of vitamin B12 tended to be lower, and biochemical status was lower in the South Asians than the other ethnic groups. Vitamin B12 bioavailability from milk is good unless heated, a process that destroys considerable amounts of the vitamin [2]. This may be a problem for South Asians, as there are reports of widespread heating of milk in Indian cuisine [38]. The last dietary factor to be assessed was fruit and vegetable intake. Given that fruits and vegetables do not contain vitamin B12, we tested whether meeting the guidelines of five or more servings a day negatively impacted serum vitamin B12 concentrations, hypothesizing that a large intake of fruit and vegetables could displace vitamin B12-containing animal products. This did not appear to be the case, as in multiple regression, fruits and vegetables were not predictive of serum vitamin B12 concentrations. This is consistent with a lack of correlation between fruit and vegetable intake and serum vitamin B12 status in adult women [39]. Our data complement those presented previously obtained for the entire New Zealand Adult Nutrition Survey sample, for which milk was reported to be the largest single contributor of vitamin B12 intakes when major food groups were sub-classified into their components [40]. For example, dairy foods were sub-classified into milk, cheese, and other dairy products, while meat was sub-classified into beef, poultry, lamb, pork, etc. Despite the prominence of milk as a contributor of vitamin B12 intake in the overall sample, the strongest predictors of serum vitamin B12 concentration in our analyses were ethnic groupings and meat consumption (combined intake of all types).

A particular strength of this study was the use of robust dietary assessment methodology coupled with a dietary habits questionnaire, which provided insight into the dietary factors associated with serum vitamin B12 status. How generalizable these findings are to the wider South Asian community living in New Zealand is uncertain, because we did not ascertain the length of time participants had been in the country, or indeed whether they were first- or subsequent-generation residents. The length of residence could influence vitamin B12 status, because a migratory effect has been found in which Indians living in the United Kingdom had higher vitamin B12 status than a matched cohort living in their home villages in India [41]. Nevertheless, the vitamin B12 status of the United Kingdom dwellers was still relatively low, at 240 pmol/L. Another limitation was the small sample size of the South Asian group, particularly of those in the older age brackets. As vitamin B12 status tended to decline with advancing age, an underrepresentation of older South Asians may have produced an underestimate of the overall prevalence of vitamin B12 deficiency in this population subgroup. Nevertheless, our data are consistent with a wider body of evidence indicating that South Asians living in various countries are at risk of vitamin B12 insufficiency or deficiency. It would be helpful for health agencies around the world to be aware of this potential for compromised vitamin B12 status in migrant South Asian communities, particularly in the identification of individuals in at-risk groups, such as the elderly and young women capable of childbearing, as well as those who do not eat meat.

## 5. Conclusions

In multi-ethnic New Zealand, we found that the Māori and Pacific and East and South-East Asian groups had the highest vitamin B12 status, followed by New Zealand European and South Asians. The group most at risk of compromised vitamin B12 status was South Asian, a group comprising people originating from the Indian subcontinent. The biochemical data were consistent with relatively low dietary intakes of red meat reported among the South Asian group.

## Figures and Tables

**Table 1:** Characteristics of participants.

Characteristic	New Zealand European	South Asian	East & South-East Asian	Māori & Pacific	Total
Total sample (*n*)	3001	124	125	1422	4672
Sex (%)					
Male	44.4	50	38.4	42.4	43.8
Female	55.6	50	61.6	57.6	56.2
Age (year) *	51.1 (24.0) ^a^	35.5 (17.7) ^bd^	33.8 (18.8) ^bc^	39.6 (16.1) ^d^	46.7 (22.4)
BMI (kg/m^2^) *	27.0 (5.5) ^a^	25.4 (4.6) ^b^	23.2 (3.7) ^b^	31.9 (7.8) ^c^	28.3 (22.4)

* Values are expressed as mean (standard deviation) standardized to the population. Differences among groups were assessed using one-way ANOVA with Bonferroni multiple-comparison test. Values with different superscript letters within a row are statistically significantly different. BMI on sub-sample: New Zealand European *n* = 2891, South Asian *n* = 121, East and South-East Asian *n* = 121, Māori and Pacific *n* = 1339.

**Table 2:** Vitamin B12 status and intake by regional ethnic grouping and sex.

Characteristic	New Zealand European		South Asian		East and South-East Asian		Māori and Pacific		Total	
	*n*		*n*		*n*		*n*		*n*	
Intake (µg/day)										
Total *	3001	4.1 (3.8, 4.3) ^a^	124	3.0 (2.50, 3.60) ^a^	125	4.5 (3.7, 5.3) ^ab^	1422	5.1 (4.7, 5.5) ^b^	4672	4.4 (4.2, 4.6)
Male	1333	4.9 (4.5, 5.3)	62	3.9 (2.90, 4.90)	48	4.6 (3.3, 6.0)	603	6.2 (5.4, 6.9)	2046	5.2 (4.9, 5.6)
Female	1668	3.4 (3.2, 3.6)	62	2.2 (1.80, 2.50)	77	4.4 (3.4, 5.3)	819	4.4 (4.0, 4.8)	2626	3.7 (3.5, 3.9)
Intake < 2.0 µg/day (%)	945	31.4%	55	44.4%	40	32.0%	397	27.9%	1437	30.8%
Serum B12 (pmol/L)										
Total *	2143	324 (317, 330) ^a^	68	282 (251, 312) ^b^	67	426 (386, 466) ^c^	733	425 (412, 437) ^c^	3011	350 (344, 356)
Male	945	318 (309, 327)	34	269 (223, 316)	22	380 (338, 423)	318	435 (418, 452)	1319	346 (338, 354)
Female	1198	328 (318, 338)	34	293 (253, 334)	45	448 (393, 504)	415	416 (399, 434)	1692	353 (344, 362)
Vitamin B12 <148 pmol/L (%)	70	3.3%	2	2.9%	0	0.0%	6	0.8%	78	2.6%
Vitamin B12 148–221 pmol/L (%)	439	20.5%	28	41.2%	2	3.0%	57	7.8%	526	17.5%
Vitamin B12 >221 pmol/L (%)	1634	76.2%	38	55.9%	65	97.0%	670	91.4%	2407	79.9%

Values are expressed as mean (95% confidence interval); *n* = Number of subjects; * Differences among groups for the total sample were assessed using one-way ANOVA with Bonferroni multiple-comparison test. Values with different superscript letters within a row are statistically significantly different.

**Table 3:** Mean (95% confidence interval) serum vitamin B12 concentration (pmol/L), according to age range and ethnicity.

Ethnic Origin	*n*	15–64 Years	*n*	65–75 Years	*n*	>75 Years
New Zealand European	1183	342 (334, 351)	478	304 (292, 317)	475	297 (280, 313)
South Asian	62	286 (255, 318)	5	247 (172, 323)	1	150 Not applicable
East & South-East Asian	70	423 (386, 461)	3	338 (135, 541)	5	307 (279, 335)
Māori & Pacific	650	423 (410, 436)	63	473 (421, 525)	16	317 (254, 381)
Total *	1965	370 (363, 377)	549	323 (310, 337)	497	300 (283, 316)

* *p* for trend across the age groupings.

**Table 4:** Vitamin B12 intake and status of participants, according to responses to the Dietary Habits Questionnaire.

Foods	*n*	Intake (µg/Day)	*p*-Value	*n*	Serum (pmol/L)	*p*-Value
Red meat						
Yes	4427	4.5 (4.3, 4.7)	0	2871	352 (346, 358)	0.005
No	269	2.7 (2.4, 3.0)		167	315 (290, 339)	
Milk						
Yes	4341	4.4 (4.2, 4.6)	0.118	2801	349 (343, 355)	0.46
No	198	3.7 (3.0, 4.3)		139	339 (307, 370)	
Fruit & vegetable						
≥5+ a day	2440	4.2 (3.9, 4.5)	0.058	1708	344 (336, 352)	0.078
<5 a day	2099	4.6 (4.3, 4.8)		1232	355 (346, 364)	
Alcoholic drinks *						
≤1/month	1329	4.2 (3.9, 4.6)	0.142	840	351 (340, 362)	0.725
≤4/month	921	4.5 (4.1, 4.9)		580	347 (334, 360)	
≤3/week	667	4.4 (4.0, 4.7)		433	352 (339, 365)	
≥4/week	818	4.9 (4.2, 5.6)		611	327 (313, 341)	

Values are expressed as mean (95% confidence interval); * One standard drink in New Zealand contains 10 g alcohol. The frequency of intake is unknown for some participants, who either did not answer or considered the question as not applicable to them; *n* = number of participants.

**Table 5:** Proportion (%) and 95% confidence interval of vitamin B12 intake from food among ethnic groups.

Food Group	New Zealand European	South Asian	East & South-East Asian	Māori & Pacific
Dairy	32.6 (31.6, 33.5) ^a^	43.9 (37.3, 50.4) ^c^	21.5 (17.6, 25.4) ^b^	21.9 (20.6, 23.1) ^b^
Meat	29.8 (28.7, 30.8) ^a^	20.7 (15.2, 26.1) ^c^	31.7 (26.5, 36.8) ^ab^	37.6 (35.9, 39.3) ^b^
Eggs	6.3 (5.7, 6.8)	5.2 (2.7, 7.6)	6.6 (3.7, 9.5)	7.6 (6.7, 8.4)
Fish	8.4 (7.7, 9.2) ^a^	11.6 (6.8, 16.4) ^ab^	11.8 (7.9, 15.8) ^ab^	13.2 (11.8, 14.7) ^b^
Bread based	5.9 (5.3, 6.5)	4.2 (1.5, 6.9)	5.5 (2.9, 8.1)	6.1 (5.2, 6.9)
Various other	17.0 (16.2, 17.9) ^a^	14.4 (10.3, 18.5) ^ac^	23.0 (18.3, 27.7) ^b^	13.7 (12.5, 14.9) ^c^

One-way ANOVA with Bonferroni adjustment, values with different superscript letters within a row are statistically significantly different, *p* < 0.05. Dairy comprises milk, dairy products, cheese, butter, and margarine. Meat comprises beef and veal, lamb/mutton, pork, poultry, other meat, sausages and processed meats, and pies and pasties. “Various other” is made up of all other food groups that individually provided less than 5% of the vitamin B12 intake.

**Table 6:** Relationship between serum vitamin B12 status and predictor variables using multiple regression.

Factor	Regression Coefficient (95% CI)	*p*-Value
Regional ethnic grouping	24.23 (20.37, 28.09)	<0.001
Vitamin B12 intake	5.87 (3.50, 8.23)	<0.001
Meat	27.03 (6.55, 47.51)	0.010
Age	−0.65 (−0.87, −0.44)	<0.001
Fruits & vegetables	−3.26 (−12.68, 6.16)	0.498
BMI	0.19 (−0.60, 0.97)	0.638
Sex	1.81 (−7.57, 11.18)	0.706
Milk	6.84 (−14.78, 28.45)	0.535

Multiple regression, variables other than age and sex were adjusted for age and sex using blood weight prioritized; age and sex were adjusted for each other.
